# Predicting traffic noise using land-use regression—a scalable approach

**DOI:** 10.1038/s41370-021-00355-z

**Published:** 2021-07-02

**Authors:** Jeroen Staab, Arthur Schady, Matthias Weigand, Tobia Lakes, Hannes Taubenböck

**Affiliations:** 1 German Aerospace Center (DLR), German Remote Sensing Data Center (DFD), Weßling, Germany; 2 Geography Department, Humboldt-University Berlin, Berlin, Germany; 3 German Aerospace Center (DLR), Institute of Atmospheric Physics (IPA), Weßling, Germany; 4 Department of Remote Sensing, Institute of Geography and Geology, University of Würzburg, Würzburg, Germany; 5 Integrative Research Institute on Transformations of Human-Environment Systems (IRI THESys), Berlin, Germany

**Keywords:** Environmental monitoring, Exposure modeling, Geospatial analyses

## Abstract

**Background:**

In modern societies, noise is ubiquitous. It is an annoyance and can have a negative impact on human health as well as on the environment. Despite increasing evidence of its negative impacts, spatial knowledge about noise distribution remains limited. Up to now, noise mapping is frequently inhibited by the necessary resources and therefore limited to selected areas.

**Objective:**

Based on the assumption, that prevalent noise is determined by the arrangement of sources and the surrounding environment in which the sound propagates, we build a geostatistical model representing these parameters. Aiming for a large-scale noise mapping approach, we utilize publicly available data, context-aware feature engineering and a linear land-use regression (LUR) model.

**Methods:**

Compliant to the European Noise Directive 2002/49/EG, we work at a high spatial granularity of 10 × 10-m resolution. As reference, we use the day–evening–night noise level indicator *L*_den_. Therewith, we carry out 2000 virtual field campaigns simulating different sampling schemes and introduce spatial cross-validation concepts to test the transferability to new areas.

**Results:**

The experimental results suggest the necessity for more than 500 samples stratified over the different noise levels to produce a representative model. Eventually, using 21 selected variables, our model was able to explain large proportions of the yearly averaged road noise (*L*_den_) variability (*R*^2^ = 0.702) with a mean absolute error of 4.24 dB(A), 3.84 dB(A) for build-up areas, respectively. In applying this best performing model for an area-wide prediction, we spatially close the blank spots in existing noise maps with continuous noise levels for the entire range from 24 to 106 dB(A).

**Significance:**

This data is new, particular for small communities that have not been mapped sufficiently in Europe so far. In conjunction, our findings also supplement conventionally sampled studies using physical microphones and spatially blocked cross-validations.

## Introduction

Today, noise is ubiquitous—it is prevalent in and around urban areas (see [1]) and even pervades remote protect areas [2]. Multiple studies have shown its influence on annoyance, stress and subsequent cardiovascular diseases, sleep disturbance, and further impairments [3–5], as well as impacts on animals [6] and ecosystems in general [7]. Thereby, noise emitted from infrastructures such as roads, airports or from industries is not spatially distributed equally but confined to specific areas. Consequently, noise affects the population to a varying degree depending on their place of residence and spatial behavior. In fact, multiple studies in the domain of environmental justice have found that noise exposure is particularly affecting social groups of lower socioeconomic position [8–11].

In Europe, noise pollution has received increasing societal and political attention leading among others to the establishment of the European Noise Directive (END) [12]. Noise, however, is highly complex in its spatial and temporal variability so that quantification and mapping is challenging. Actual field measurements need to be comprehensive and therefore are expensive. However, sophisticated engineering methods can be deployed to map simulated noise [13, 14]. Provided with detailed traffic information and subsequently with data describing the environment, these source–path–receiver-based simulations are known to be very accurate. In accordance with the END, this approach is deployed to generate strategic noise maps every 5 years in Europe. Amongst other parameters, these maps include *L*_den_, the yearly averaged noise estimate condensing weighted day, evening, and night periods [15], specific to individual noise emitters (e.g., road traffic) at 4 m above the ground. Notwithstanding its merits though, the END has limitations as well—particular for consecutive exposure studies on regional or even national scale. Road noise, for example, only needs to be mapped in urban agglomerations with more than 100,000 inhabitants ([12] art.3 sec. k) and for rural areas along major roads with more than 3,000,000 vehicles per year ([12] art.3 sec. n). Therefore, predominantly no noise data are available for smaller urban areas and other roads in peripheral areas. In regards to environmental health equity though, the incomplete data bases of rural areas distort direct comparisons of affected populations [16]. Also, where a large city physically grew outside its administrative boundaries [17], areas not mapped *L*_den_ > 55 dB(A) correspond to the two separate END sections k) and n) of article 3 and thus have different semantics as well: inside the boundaries, these areas represent *L*_den_ < 55 dB(A), whereas in the suburbs, these areas actually were not mapped at all. The epidemiological analysis of exposure to noise therefore often remains spatially partial, or too aggregated.

For such studies, we aim at leveling data inequities and search for a scalable noise mapping approach. In, but particularly outside Europe, epidemiologists extrapolate microphone measurements using kriging (e.g., [18]) and land-use regressions (LURs) [19–26]. Both approaches are relatively inexpensive but comparing them, Xie et al. [19] have found LUR models produce better results. Thereby LUR, initially developed to assess the exposure of air pollution [27], integrate multiple spatial predictors into a statistical model. After preprocessing (i.e., log-transformations, feature selections, etc.), a statistical model is fitted, which conclusively can be used to estimate noise exposure. Building on the fact that Aguilera et al. [21] found high correlations between in situ noise measurements and END compliant noise maps, we utilize the latter *L*_den_ to learn a LUR model and transfer the encoded information to surrounding areas. Thereby, likewise referring to virtual microphones, we investigate the implications of different sampling schemes, i.e., sizes and localizations, by repeating the procedure 2000 times. With this, overall 185,000 samples were drawn and tested for quantifying statistical uncertainties. In particular, as spatial autocorrelation is a distinct matter for local acoustical phenomena, we extend the investigations of Liu et al. [26] by introducing two structured cross-validation approaches [28] for LUR. Blocking samples by specific classes related to urban morphology as well as by administrative districts respectively, a comprehensive set of spatially independent cross-validations allows for assessing the transferability of the model to new, unseen areas. Discussing those last two parameters, sampling scheme and cross-validation, contributes to the development of LURs in general and outside Europe as well. For our study though, the conjunction provides comprehensively benchmarked noise predictions in particular. As a result, a detailed noise map presents continuous exposure levels for peripheral areas not included in the END obligation yet.

## Material and methods

**Fig. 1:**
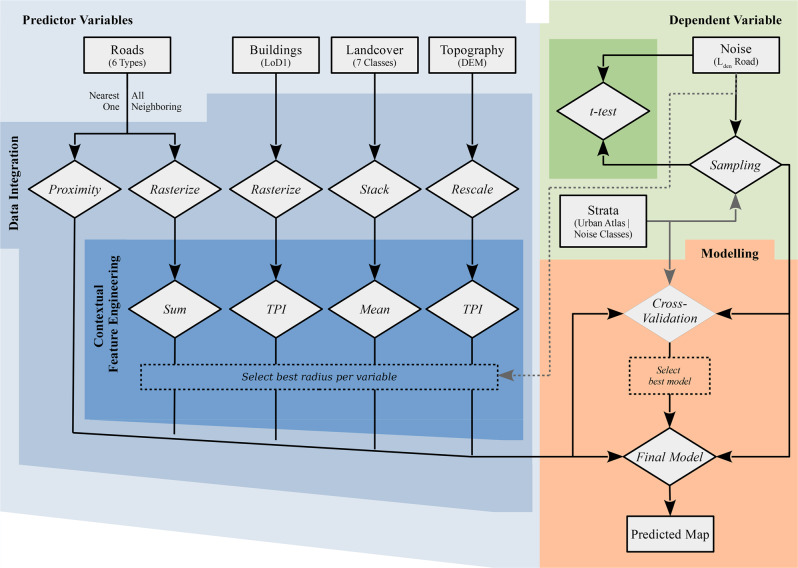
Flowchart grouping data (rectangles) and methods (rhombuses) into main methodical steps (colored backgrounds). Black lines illustrate data flow in general, while gray lines denote auxiliary information needed within individual steps only. Dotted elements denote selection processes.

After introducing the study area first, we start with compiling the model’s independent predictor variables (highlighted blue in Fig. 1). In order to represent road traffic noise emissions and respective soundwave interactions with the environment, four different inputs are considered—road infrastructures, built-up structures (LoD1), and the natural environment in terms of land cover (LCC) and topology (DEM). In a preprocessing step, these data are integrated matching the 10 × 10-m resolution commonly obliged in European noise studies. As noise may travel over large distances, contextual effects are embraced at eight moving window radii ranging from 12.5 to 1600 m. After the most relevant radius per variable is selected, the predictors are embedded into the modeling. Analogous, simulated noise data are sampled (highlighted in green), later also referred to as virtual microphones. Among four investigated sampling schemes, two stratified approaches required urban land-use categories and noise classes as stratum, respectively. The sampled data sets are described statistically (*t*-test), before being related to the predictor variables (highlighted orange). During various cross-validations, the parameters are assessed. Last but not least, both the data are used for training a final model, which eventually is applied to a larger area.

### Area of interest

In this study, we exemplify the emission and propagation of traffic noise in and around Koblenz, a German city with 113,844 inhabitants living on 105 km^2^ [29]. The city of Koblenz represents an excellent case for training the model because of its different types of built structures, the heterogenous natural environment and topography, a diverse road network, and spatially nuanced noise patterns. Id est, its administrative boundaries embrace different structural features from dense built-up morphologies to low-density rural areas, and from flat areas to steep fluvial shaped flanks by the meandering rivers Rhine and Mosel (see Fig. S1).

Further, Koblenz is surrounded by the travel region Rhine valley, defined by the German statistical office (Destatis) and the Federal office for building and regional planning (BBSR). This 924 km^2^ large geographic entity is situated along the river Rhine, connecting four cities and 56 municipalities with a total population of 773,071 [29]. We chose this area for deploying the final LUR model, as it surrounds the city of Koblenz and shares morphological features with it. That are the suburban structures along the northern river loop (e.g., Vallendar, Bendorf, Neuwied, etc.) in particular, as well as agrestic patterns opening out south. Further, it is interesting to note, that due to major roads most often located parallel to the river and thus outside this touristic appealing region, noise data are rarely available.

### Sampling noise simulation data

**Fig. 2:**
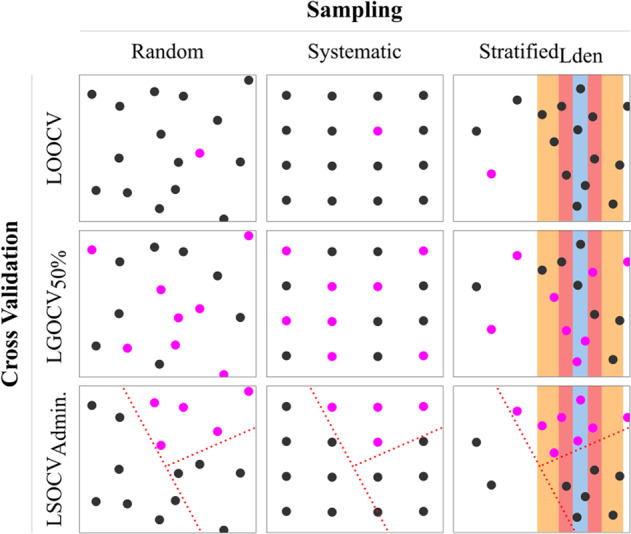
Illustration of sampling schemes and different cross-validations concepts. Highlighted dots (purple) represent samples being left out for an individual cross-validation iteration. Colored background illustrates noise levelsused as strata. Dashed red lines depict administrative borders used for blocking the cross-validation.

Due to the expensive nature of noise measurement sampling, the sampling schemes applied in other studies are more often stratified [19–22, 25, 26, see also Table S1] than random [24]. This is not surprising, as stratified sampling is known to be more cost-effective [30]. According to our knowledge, a systematic sampling approach has not been deployed yet. In both, systematic and random sampling, the sample units are drawn independently from each other with an equal probability such as they can represent the complete data set best (see first two columns of Fig. 2). Particular at smaller sample sizes though, both approaches can lead to relatively large gaps in the sampled area as well. In related literature, sample sizes range from 40 (Girona in [21]), up to 729 (across five cities in [26]). As the importance of locating the samples was stressed from the very beginning of noise-related LURs [15], we simulate its effects in this study with 2000 virtual field campaigns. Thereby, our sampling experiments systematically vary sampling scheme, sample size, and random influences. We considered random, systematic, and two variants of stratified sampling schemes aiming for a comprehensive representation of noise levels. The latter, stratified sampling, is to ensure that the whole population is represented well at reduced collection costs [30]. We reproduce the approach of Chang et al. [25], stratifying based on land-use categories, using the 22 different LU/LC classes defined by the European Urban Atlas [31] as strata. Analogous, as the actual noise data are already prevalent, we also stratified samples based on 5-dB(A) increments (illustrated in column three of Fig. 2). Further, investigating uncertainties due to the sample size, *N* was increased from 50, 100, 200, 500 to 1000 for each sampling scheme. These *N*s are akin to previous studies (see Table S1) and beyond. Regarding stratified sampling though, the scarce outer classes may lead to an actual smaller *N* eventually sampled (hereafter referred to as *N*_sampled_). To account for bias of random sample generation, we iterate each experiment with 100 different random number seeds. In case of regular sampling, this seed refers to an offset in the sampling grid.

As reference, we considered EU compliant noise simulations for our virtual campaigns. The local authorities of Koblenz provided us with the simulation results of 2017 (see center Fig. S2). Such maps, fulfilling the END, are produced combining high resolution input data such as traffic information and built-up inventory with ray-tracing propagation models [13, 14]. The engineering’s software output has a spatial resolution of 10 × 10 m, the continuous pixel values depict the simulated road traffic noise *L*_den_. Thereby, the *L*_den_ ranges from 12.8 to 88.3 dB(A), with a mean of 51.0 dB(A) and a standard deviation of sd = 11.1. The advantage of using END conform noise maps instead of actual in situ measurements is, that they are source-specific (i.e. road traffic noise) and they provide annually averaged sound pressure levels, which both are the basis for political decision making. Also, the 1.05 million pixels of our study area allow for investigating sampling effects comprehensively, which is important in order to assess the transferability of the model. Due to a heterogeneous infrastructure network as well as different topographic, built-up and natural morphologies, the sound pressure levels, however, are not uniform (see map Fig. S2 or Table S2 for descriptive statistics along all 30 districts, respectively. Table S3 for descriptive statistics with respect to the Urban Atlas), but structured spatially. Two very loud and long-range hearable motorways are allocated in the north-east, affecting large proportions of the adjacent districts. From there, a trunk road runs through the city center in a southerly direction, before finally connecting the eastern parts of the city over the Rhine river. Primary roads are located along the river banks and one (B327) meandering uphill South-southwest. Here it is well depicted, that the scope of noise propagation depends on the surrounding natural environment as well. Zooms b&c in Fig. S2 highlight local effects by highly built-up urban morphology and the topography.

The sampled *L*_den_ sound pressure levels are considered virtual microphone measurements. In order to assess the sampling impacts statistically, the representativity of each of the 2000 virtual field campaigns is assess by aggregating their mean and standard deviation first. Then, they were related to the total population using a two-sided *t*-test assuming the variances to be equal.

### Predictor variables

In general, LUR predictors in noise-related studies vary from physical road infrastructures [19–26], traffic information [21, 22, 24–26], surrounding buildings [20–24], land-use/-cover patches [19–26], vegetative indices [20, 22, 23, 26], and amongst weather data [25] and topography [26] to any other auxiliary information hypothesized to affect the response variable. Based on this literature and data review (see also Table S1), we inherit the most common features and, aiming for large-scale reproducibility, prioritize publicly-accessible data sources throughout the study. To incorporate contextual effects like the arrangement of streets, built-up density, and fraction of green spaces, moving windows were utilized. The respective aggregation functions (sum, mean, etc.) depend on the variable and their respective geographic rational, which is laid out below. Regarding the radii, preceding studies did not agree on a standardized and commonly accepted rule for defining them. Nevertheless, the applied radii generally range between 50 and 1000 m and also some particular scales were already used more often (highlighted bold in following enumeration). In relation to the logarithmic behavior of noise attenuation, we thus used systematically scaled radii of 12.5, **25**, **50**, **100**, **200**, 400, 800, and 1600 m. Then, following the descriptions of Ragettli et al. [22] and Liu et al. [26] the most relevant radius per feature was selected a priori. To do so, a bivariate linear model was fitted for each available variable. The most relevant radius per variable was chosen based on the smallest root mean square error (RMSE). Thus, together with six road proximity features, eventually 21 features were considered.

#### Road infrastructure

In this study, we focus on traffic noise emitted along roads. In consequence, the road network and the proximity hereto are essential. We take the spatial layout of the streets from OpenStreetMap (OSM) data. Since data on traffic counts were not available, we considered the assorted functional road types Motorway, Trunk, Primary, Secondary, Tertiary and Residential as proxies for traffic volume capacities and speed limits. We tested the suitability of this metadata by investigating the *L*_den_ values at the road center. A subsequent ANOVA tests for significantly different noise levels between the six road types. Then, being coded as dummy variables [32], their effects were added to the model for each road type separately. Hereby we distinguish two effect types—proximity and cumulated road length. In both cases, tunnels were excluded throughout the study [21].

At a scale of 10 × 10-m resolution, the proximities to roads were derived, such as the digital numbers of the grid depict to distance to the nearest road in meters. We consider this raster-based approach, as conducted by Harouvi et al. [24], more sensitive than vectored buffer rings [19–23, 25] and therewith justify the additional computational resources needed. Along these gradients, with increasing distance to the source, sound levels are known to decline on a logarithmic basis [33]. Consequently, the feature space was log-transformed.

In addition, for each location, all neighboring roads were considered. This allows cumulating multiple road exposures such as at intersections and multilane roads. To do so, we first rasterize the input features, where the value of each cell represents the subjacent length of a road multiplied by its number of lanes, as it is recommended in the European Good Practice Guide [34]. Where the latter attribute was not available, it defaults to one. Then, the cumulated road length was summarized [19–23, 25, 26] using the systematically scaled moving windows defined above.

#### Urban morphology

Buildings and their corresponding volume are also essential for sound propagation [35–37]. On the one side, their emission facing façade reflects the sound. This is very well exemplified by dense street canyons, which are known to increase noise levels [38]. Vice versa, on the side of the buildings facing away from the road, sound levels are significantly lower. To include urban morphologies, building footprint and height data are added to the model from the nationwide Level-Of-Detail-1 data set by the Federal Agency for Cartography and Geodesy [39].

Then, using the moving windows defined above, the neighborhood was integrated to the list of predictors by averaging the mean built-up height. Similar to the topographic position index (TPI) introduced by Weiss [40], the values were normalized by subtracting the built-up height of the central pixel. Positive values depict superior locations, while negative values represent locations that are lower than their surroundings (valleys). Therefore, these values correspond to the volume available for sound propagation.

#### Natural environment

Analogous to the built environment, the surrounding topography has an important influence on sound propagation [41]. Therefore, the EU-DEM [42] was rescaled to the 10 × 10-m resolution using bilinear interpolation. The respective TPI was derived at the above-defined moving window radii as well.

In addition, propagating sound waves interact with the ground surface [43]. While plain and solid surfaces such as water bodies and some artificial material have reflecting acoustical properties, other, soft, or porous material have absorbing effects [41, 44]. In the case of vegetative cover, experiments comparing noise propagation in areas covered with trees against such covered with grass have shown that this effect is proportional to biomass [45, 46]. In our study, we represent the earth’s surface by a remote-sensing-derived land-cover classification [47]. Computed from a multi-temporal Sentinel-2 data set using Land-Use/-Cover Area frame Survey reference samples, this publicly available product with an overall accuracy of 93.1% has the same spatial properties as the reference units defined above. It includes seven land-cover classes expected to be important for noise mapping: artificial land, open soil, water areas, and detailed information about vegetative biomass and its seasonality (low perennial, high perennial, low seasonal, and high seasonal vegetation). As a part of the data harmonization process, each land-cover class is treated separately [32]. The moving windows computed the respective land-cover fraction for each given neighborhood radius in percent.

### Modeling

To test the potentials and limitations of our various sampling experiments systematically, we have chosen linear least squares regressions. These are most commonly used in this thematic context [19, 21, 23–25]. In addition, their computational complexity is manageable. This is relevant for both, repetitive training of our 2000 virtual field campaigns, and prediction. With respect to the 10 × 10-m resolution, it is crucial that the model can be deployed to larger areas of interest. Keeping the focus on sampling artefacts, we selected a simple and consistent model implementation and ruled out variability between the experiments by not considering forwards- (as in [21–23, 25]) or backwards-selecting approaches [19, 20, 24, 26].

The model performances are assessed using five complementing variations of cross-validations and three accuracy measures. Cross-validation is a common approach aiming for a statistically independent evaluation, particularly interesting at small sample sizes. Leaving *n* random samples out, the model is fitted excluding these *n* samples, and evaluated against them in a subsequent step (c.f. rows in Fig. 2). Herewith an independent measure is available in order to quantify the transferability of the model. Typically (as in [20–22, 25, 26] *n* is one (hereafter revered to as LOOCV) or (as in [19, 24, 26]) a fraction, e.g., 10% random samples (hereafter referred to as leave-group-out LGOCV_10%_). Similar to Liu et al. [26], in this study, we implemented LOOCV, LGOCV_10%_, LGOCV_25%_, and LGOCV_50%_. However, especially when the data are structured, e.g., by landscape, spatial autocorrelation can jeopardize the central assumption that training and evaluation data are independent, such as no conclusions regarding overfitting can be drawn [28]. For such applications, Roberts et al. [28] recommended blocking (although the division do not need to be rectangular) the cross-validation samples accordingly. That is, a model is trained on one proportion of the city and then tested in a new, unseen area. As the spatial transferability is of particular interest in our study, we consider two approaches: LSOCV_Admin_ leaves out one of the 30 spatial districts of Koblenz at a time and second, LSOCV_Urb.Atl._ validates the transferability by blocking samples based on the urban settlement structures. This is achieved by utilizing polygon geometries of 22 different LU/LC classes from the European Urban Atlas data set [31] as blocks.

We evaluate the models’ accuracies by calculating the coefficient of determination (*R*^2^), root mean squared error (RMSE) and mean absolute error (MAE). During the cross-validations, the accuracy metrics were summarized into mean and standard deviation. Further, for each sampling experiment we fit an overall model including all samples. Although the accuracy metrics for this model do not indicate its transferability, it is presumed to be the best trained one and should be deployed on new data [28]. The discrepancy between the cross-validated and overall accuracy metrics indicate the internal validity of a model [25]. According to Eeftens et al. [48], a *R*^2^ difference of <0.15 resembles a robust model. Eventually, after selecting a final model based on these evaluations, it was deployed to the neighboring communities in the Rhine valley.

## Results

### Predictor variables

#### Road infrastructure

In particular, the OSM roads have to be examined in detail for their suitability as eminent proxies. Along their spatial representation, the six individual road types differentiated in OSM indicate different traffic intensities, speed limits, and road surface properties. To ensure that this classification was an appropriate representation of the resulting noise emissions, *L*_den_ at a distance of 0 m, id est on the road itself was investigated. Despite some larger standard deviations for tertiary and residential roads, Table 1 reports that all road types have individual noise levels in terms of mean and median. This observation is empirically backed by a corresponding, highly significant (*p* < 0.001) ANOVA analyses. Although visually some overlaps exist, the subsequent Tuckey test finds significant (*p* < 0.001) difference between all road types.

**Table 1:** Summary of road *L*_den_ for six OSM road types on roads at 0-m distance.

	Min.	1st quartile	Median	Mean (Sd.)	3rd quartile	Max.
Motorway	66.90	82.90	85.90	83.90 (4.93)	86.90	89.10
Trunk	55.40	73.50	77.40	76.37 (5.16)	79.40	86.30
Primary	57.30	70.20	73.10	73.14 (3.68)	75.70	83.50
Secondary	10.50	66.50	70.00	68.00 (7.92)	72.20	88.90
Tertiary	18.60	48.60	67.20	60.13 (14.10)	70.80	84.20
Residential	22.60	46.20	50.40	51.44 (8.39)	55.30	84.20

Starting with a distance of 0 m, the proximity with respect to residential roads ranged up to 2 km, respectively, 12 km as to motorways (see log10-transformed values in Table 2). As the proximity to roads showed an exponential relationship toward *L*_den_, a log transformation was required [21, 24, 25]. After the preprocessing, the Shapiro–Wilk test certifies a normal distribution for all individual road types (*p* < 0.001).

**Table 2:** List of selected predictors, their metadata, their range, and their RMSE in bivariate linear models explaining *L*_Den_ used for selecting the appropriate scale.

Source	Feature	Attribute	Units	Min.	Max.	R^2^	RMSE
OSM	Motorway	Proximity	log(M)	0	3.99	0.22	9.842
		Length_800_	km	0	17.61	0.17	10.148
	Trunk	Proximity	log(M)	0	3.744	0.13	10.391
		Length_400_	km	0	9.34	0.14	10.318
	Primary	Proximity	log(M)	0	3.67	0.03	10.963
		Length_100_	km	0	1.76	0.05	10.836
	Secondary	Proximity	log(M)	0	3.65	0.08	10.694
		Length_1600_	km	0	16.99	0.09	10.658
	Tertiary	Proximity	log(M)	0	3.48	0.05	10.887
		Length_1600_	km	0	28.00	0.04	10.906
	Residential	Proximity	log(M)	0	3.30	0.02	11.017
		Length_800_	km	0	21.67	0.02	11.014
BKG	LoD1	TPI_800_	/	−1.77	44.20	0.00	11.138
Copernicus	DEM	TPI_1600_	/	−114.62	121.66	0.04	10.932
Weigand et. al. [47]	Artificial land	Mean_800_	%	0	94.28	0.11	10.535
	Open soil	Mean_1600_	%	0	1.32	0.06	10.815
	High, seasonal veg.	Mean_800_	%	0.25	96.67	0.19	10.040
	High, perennial veg.	Mean_1600_	%	0.01	16.96	0.15	10.247
	Low, seasonal veg.	Mean_1600_	%	0.04	72.53	0.10	10.582
	Low, perennial veg.	Mean_800_	%	0	66.01	0.04	10.909
	Water areas	Mean_400_	%	0	3.99	0.02	11.015

#### Selected variable radii

Further, Table 2 presents the other selected variables, as well. We obtain multiple neighboring contexts for each predictor. Following the procedure of Ragettli et al. [22] and Liu et al. [26], we identified the most appropriate radii by the lowest RMSE in bivariate models. The subscripts depict the selected moving window size for each variable. It turned out that the influence range on the resulting sound field lies between 100 and 1600 m, respectively.

### Sample localization

**Fig. 3:**
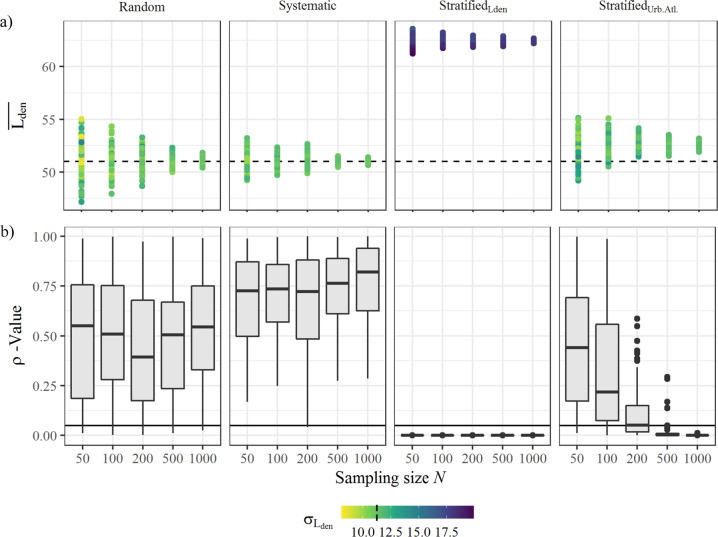
Descriptive statistics on sampled data, separated by sampling scheme (vertical facets) and size (*X*-axis). **a** Depicts mean (*Y*-axis) and standard deviation (color) of Lden. Dashed lines reference to complete data set. **b** Shows *p* value of the consequent Shapiro–Wilk test (*Y*-axis). Solid line refers to 5% confidence interval.

By permuting the four individual sampling schemes at five sample sizes and reproducing with 100 different random number seeds, a total of 2000 independently sampled data sets were produced. As stated before, the utilized noise input values had a mean of 51.0 dB(A) and a standard deviation of 11.1. In comparison thereto, not all sample sets comply. Figure 3a depicts that in general the magnitude of deviations highly depends on random effects, i.e., the seed. Naturally, at larger sample sizes, this effect alleviates. Despite that, the sample schemes show two distinct characteristics: in general, random and systematic sampling both represent the total population well in terms of comparable means and standard deviations. This is empirically backed by high *p* values (Fig. 3b). Using a systematically gridded sampling scheme, all but two sets (*N* = 200, seed = 83 and 84) exemplify the mapped noise representatively (*p* > 0.05). Stratified sampling though, particularly stratified_Lden_, returns higher *L*_den_ values. The balanced sampling based on urban morphology, stratified_Urb.Atl._, though tends to just slightly overestimate *L*_den_. The two-sided *t* test attests significant differences (*p* < 0.05) at larger sample sizes only, replicating a well-known issue of *p* values [49].

The integration of these 2000 sample experiments with the aforementioned predictors results in one corresponding LUR model each. To compare them, the three accuracy measures are summarized into mean, standard deviation, as well as the 5% and 95% percentile as a dependency of sample size and sampling scheme in Table S4. Thereby, unaffected by the sampling scheme, two trends were related to the extent: first and congruent to the aforementioned observations, the standard deviation was inversely correlated to sample size. Second, the overall accuracy decreased asymptotically for larger sample sets. This holds true for *R*^2^ where values closer to 1 are favored, as well as for RMSE and MAE where small values indicate a good fit. Nevertheless, the *R*^2^ of both stratified sampling approaches did so on a higher level, i.e., the *R*^2^ of stratified_Lden_ not falling below 0.78, respectively, 0.74 stratified_Urb.Atl,_, RMSE and MAE certify statified_Lden_ larger residuals though. These observations were backed by highly significant MANOVAs (*p* < 0.001 for both, sampling scheme and size) on *R*^2^, RMSE, and MAE. The subsequent Tukey tests point out that there was a significant difference between all parameters except increasing the sample size from 500 to 1000 observations (*p*_*R*²_ = 0.19, *p*_RMSE_ = 0.80, *p*_MAE_ = 0.97).

### Cross-validations

**Fig. 4:**
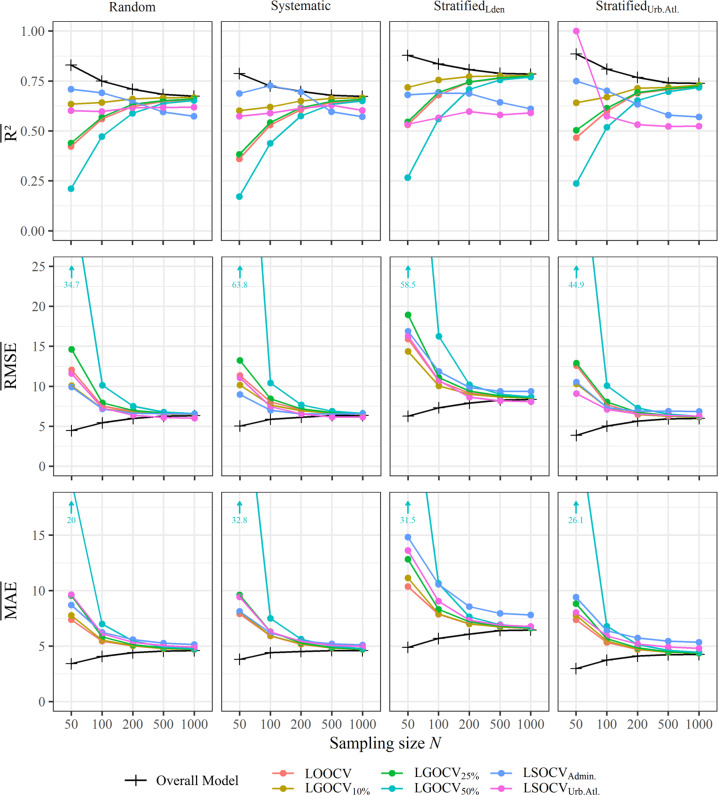
Comparing accuracy measures of overall models (black) cross-validations (colored), separated by sampling scheme (vertical facets) and size (*X*-axis). With respect to R², RMSE and MAE, each point condenses the means of 100 repetitions.

Examining the transferability of a model to unseen samples (Fig. 4), the four randomized approaches perform notably different to the two spatially independent LSOCV concepts. Id est, their cross-validated accuracy measures converge antipodal to the overall models. Eventually, at 1000 samples, the difference between the overall *R*^2^ (black lines in Fig. 4) and its cross-validated pendant (colored lines in Fig. 4), fell below the level of 0.15 (specified by 25 and 48) for all but the two LSOCVs at both stratified sampling schemes. Coefficients of determination retrieved using LSOCV_Admin._ were relatively high at lower sample sizes, but then drastically decreased at sample sizes >200. In contrast to this, the mean *R*^2^ of LSOCV_Urb.Atl._ remained steadily within a range of 0.55 and 0.64, but showed obtrusive values at *N* = 50 for the stratified sampling scheme based on the same strata. Considering the averaged RMSE and MAE, LGOCV_50%_ stands out in particular. However, no differentiating trends could be observed between spatial and conventional cross-validation approaches.

### Final model

In our geographical application, we aim to model our complete test area. For this, we select a specific model. In order to minimize random effects, such as discussed above, only the largest sample sizes were considered (*N* = 1000). Further, aiming for a high accuracy, stratified_Urb.Atl._ was selected. Although the respective *R*^2^ tends to be marginally lower compared to stratified_Lden_, the significant smaller residuals depicted in the RMSE and MAE outweigh these considerations. At these settings, the overall *R*^2^ was 0.702, adjusted *R*^2^ 0.696, respectively. All accuracy metrics are presented in Table 3.

**Table 3:** Accuracy metrics of final model (sampling = stratified_Urb.Atl._, *N* = 1000, seed = 98).

	*R*^2^	RMSE	MAE
Overall model	0.702	6.25	4.24
LSOCV_GADM_	0.592	6.92	5.30
LSOCV_UA_	0.536	6.27	4.74
LOOCV	0.683	6.46	4.35
LGOCV_10%_	0.707	6.48	4.41
LGOCV_25%_	0.680	6.53	4.41
LGOCV_50%_	0.678	6.53	4.43

In particular for subsequent map interpretation and decision making, it is of interest to assess the parameters of the final model. Starting with describing the estimated intercept, the linear regression model assumes a very high *L*_den_ of 130.78 dB(A). Subtracting thereof, the regression terms regarding the log-transformed proximity to all but residential roads had negative estimates. As can be seen in Table 4 though, these were insignificant in respect to proximity to secondary and tertiary roads. Where many motorways or tertiary roads were in close vicinity, *L*_den_ is lower; in contrast thereto, an accumulation of the other road types comparatively increases noise levels. Contemplating the other variables, not related to traffic noise emissions, a small but highly significant (*p* < 0.001) estimate of −0.04 for the topographic TPI_1600_ indicates that increased noise levels can be found in valley locations. Analogous, the negative estimate (*β* = −0.218) regarding TPI_800_ for buildings is congruent to the findings of Heutschi [38], but insignificant (*p* > 0.1). Land cover [47] was a significant parameter for four out of the seven land-cover classes (*p* < 0.10): for example, low, perennial vegetation reduced *L*_den_ by −0.17 dB(A) per percentage fractional cover within a radius of 800 m, respectively, −0.09 dB(A) for high but seasonal vegetation at the same radius. The full model is presented in Table 4.

**Table 4:** Estimates of final LUR model.

Source	Feature	Attribute	Beta	Std. Error	*t* value	Pr(>|t|)	*p* value
Intercept			130.78	4.85	26.96	0.00	***
OSM	Motorway	Proximity	−11.93	0.69	−17.39	0.00	***
		Length_800_	−0.39	0.00	−2.28	0.02	*
	Trunk	Proximity	−8.81	0.69	−12.86	0.00	***
		Length_400_	0.55	0.00	2.42	0.02	*
	Primary	Proximity	−2.43	0.63	−3.86	0.00	***
		Length_100_	17.42	0.00	5.39	0.00	***
	Secondary	Proximity	−0.56	0.55	−1.01	0.31	
		Length_1600_	0.16	0.00	1.31	0.19	
	Tertiary	Proximity	−0.81	0.53	−1.53	0.13	
		Length_1600_	−0.18	0.00	−2.60	0.01	**
	Residential	Proximity	0.95	0.41	2.29	0.02	*
		Length_800_	0.235	0.00	3.17	0.00	**
BKG	LoD1	TPI_800_	−0.218	0.19	−0.93	0.35	
Copernicus	DEM	TPI_1600_	−0.04	0.01	−4.43	0.00	***
Weigand et. al. [47]	Artificial land	Mean_800_	−0.05	0.03	−1.65	0.10	#
	Open soil	Mean_1600_	5.12	1.41	3.62	0.00	***
	High, seasonal veg.	Mean_800_	−0.09	0.03	−2.95	0.00	**
	High, perennial veg.	Mean_1600_	0.12	0.12	0.98	0.33	
	Low, seasonal veg.	Mean_1600_	0.00	0.04	0.02	0.98	
	Low, perennial veg.	Mean_800_	−0.17	0.03	−5.36	0.00	***
	Water areas	Mean_400_	−0.03	0.02	−1.16	0.24	

Levels of significance: ***p < 0.001; **p < 0.01; *p < 0.05; ^#^*p* < 0.1.

To comprehend this model further, a subsequent assessment of the residuals though showed that most of the modeled values within the range of 35–75 dB(A) align well to the official road *L*_den_ (Fig. S3c). As is visualized by the width of the boxplots, this concerns the majority of pixels. The spatial representation (Fig. S3a) of the residuals depicts two distinct issues. Large positive residuals can be found along certain roads, where in officially higher noise levels occur. Conversely, large negative residuals are found in otherwise quiet canyons. Analogous, a tendency toward smaller negative residuals was found in backyards of residential blocks, indicating that it is potentially quieter here. Putting the residuals into place, we last but not least intersected them with our building model. With respect to these vulnerable areas, the supplementary histogram (Fig. S3b) shows that actually 44% of them have an error ranging between −2.5 and 2.5 dB(A) at most. In summary, we only found an exposure-specific MAE of 3.84 dB(A) here.

**Fig. 5:**
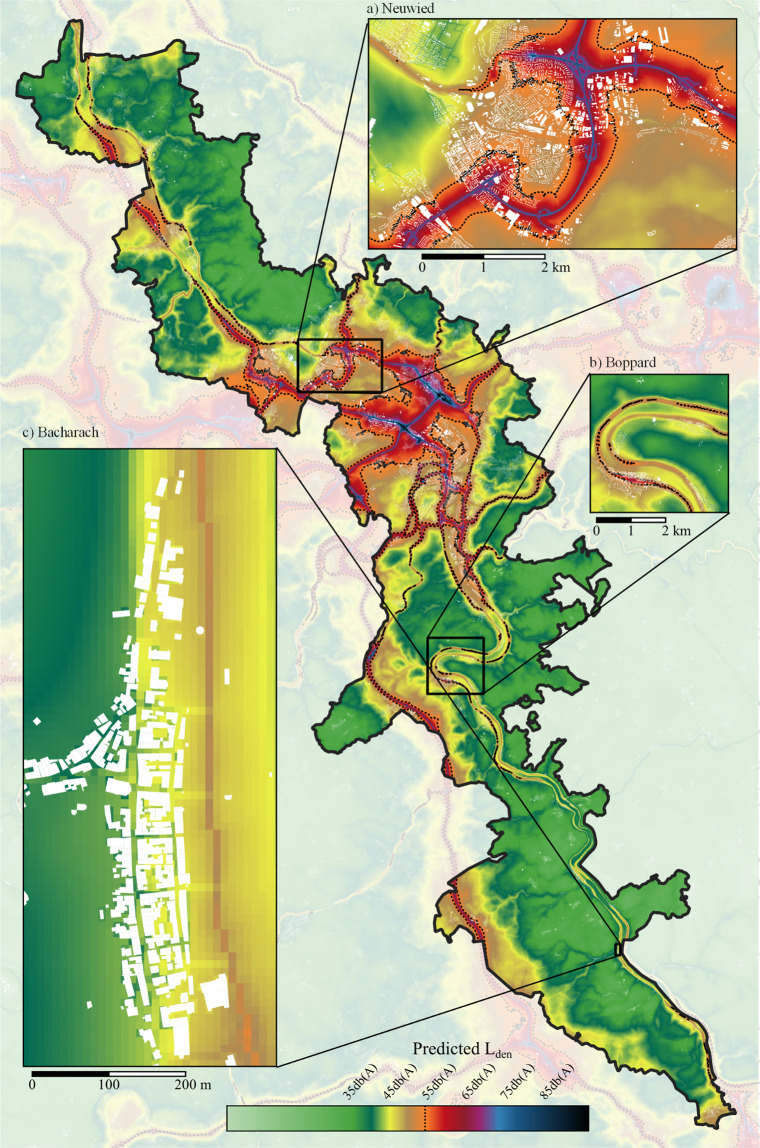
Predicted noise levels for the Rhine valley. Zooms show (**a**) highly dense suburbs and (**b**) low populated villages along the river Rheine, as well as (**c**) an exemplary detail at very low scale. Continuous colorscheme akin to DIN 18005, dotted line depicts 55-dB(A) contour, build-up areas are colored white.

The selected final model was then deployed on the urban, peri-urban, and rural regions of Koblenz and surroundings. The map (see Fig. 5) shows that 90.8% of the Rhine valley are below the threshold of 55 dB(A). With respect to exposed populations in particular, the same is true only for 79.2% of the build-up areas. An example of such is visualized for the flat and highly populated city Neuwied (Fig. 5a), bordering in the northwest of Koblenz. Moreover, our results also show that in close vicinity to the Rhine, where primary roads can be found on both river sides, such critical noise levels can be found in peripheral regions as well (Fig. 5b). Vice versa, it is interesting to see at the example of Bacharach (Fig. 5c) that existing noise preventive planning such as bypass roads are highly effective. Id est, this example illustrates very well how built-up structures can block propagating noise. However, some noise is still emitted along the village’s main road itself, particular in the northern part. But, this does not exceed the critical threshold of 55 dB(A) *L*_den_. In summary, the resulting final noise map provides valuable insights about possibly affected populations in suburban and peripheral areas.

## Discussion

In this study, we transferred noise information encoded in official noise maps to statistical models. Thereby, the very details of sampling noise, in this case evaluated using thousands of virtual microphones, and their influence was assessed. In contrast thereto, a small feature set was held constant. Overall, the model proves accurate results for a large-scale area. However, some critical aspects of our approach are to be pointed out: when subsampling END conform noise for training LURs, two issues arise: first, as LURs usually are used to extrapolate in situ noise measurements into larger areas, one may discuss training them with *L*_den_ maps. In this study, we substituted in situ noise measurements with *L*_den_ maps and could show that generally existing noise map can be predicted beyond its administrative limitations to estimate respective noise exposure levels for surrounding areas at low costs. Second, one needs to be aware that errors and methodical shortcomings of this input source will propagate. EU compliant calculation methods are configured conservatively, such that the predicted noise levels and the consecutive number of people exposed to noise both are overestimated (cf. [34]). In addition, it needs to be noted that *L*_den_ quantifies noise exposure dosage but insufficiently predicts health relevant noise annoyance [50]. Therefore, we need to be aware that a finer temporal resolution of noise levels, e.g., 10 min, 1 h, etc., or quantifying significant noise peaks is not included in the analysis, but would be interesting from an epidemiological point of view [51].

In addition, and also relevant for conventionally sampled LUR studies, important methodical aspects could be tested with this approach. Random and systematic sampling schemes both retrieve the most representative samples. However, they both were sensitive to random effects and require larger sampling sizes—probably impracticable for time and cost expensive field campaigns. Stratified sampling on the other hand is sensitive to the precise sampling scheme. Referring to sample size only, we found no significant model improvement for *N* > 500. Stochastically, 500 samples in our 105 km^2^ large area refers to a Euclidean distance of 458 m between the virtual microphones. Most probably due to wireless sensor networks [52], such dense sampling strategies appear economically feasible for limited areas. For assessments on national levels or the like, however, this still is not the case and will be difficult to achieve. Thus, END conform that predicted noise maps are an alternative.

Further, on smaller scopes, the spatial distribution of noise can be both, clustered i.e., along line sources as well as disperse. Such spatial autocorrelation is known to challenge statistical modeling and can jeopardize the assumptions of cross-validations [28, 53]. Particularly at larger sample sizes, the cross-validated accuracy measure converges with the overall models’ *R*^2^. As this effect was best observed with stratified sampling, where especially very loud samples were limited and therefore in close vicinity, we infer it being caused by spatially neighboring samples. Confirming the experiences made in ecological studies [28], the informative value of conventional leave-*n*-out cross-validations may be limited in such structured data. Only LSOCV does encounter jeopardizing the credibility of CV in regards of actually assessing overfitting. Nevertheless, the administrative blocking proved holding out circumscribed portions of the predictor space, i.e., very loud motorways in the peripheral, north eastern district of Rübenach. In such cases, the model needs to extrapolate into unknown administrative areas. The same holds true for LSOCV_Urb.Atl_, where we specifically challenged the model to transfer into unknown settlement structures. In both cases, the accuracy measures retrieved with the cross-validations actually reflect these transfer abilities. To reduce further covariations in the data introduced by proactive landscaping, likewise other spatial blocking concepts, such as rectangular, hexagonal, or radial, may be considered in future.

Aiming for an easy reproducible study design, the model focused on road-related traffic noise. Therefore, the predictor variables scope road infrastructures, urban morphology and the natural environment only. Although this variable set is relatively small, nevertheless it was capable of explaining large proportions of the overall variance. Moreover, similar to the models of Harouvi et al. [24], proximity of the traffic-related features is more significant than the cumulated sum. The negative betas of road proximity for example appear logical, as with increasing distance to an emission source, noise levels decrease. For residential roads though, most frequently found in respective residential areas, the proximity thereto was rather an indicator for quietness. A significant correlation (*r* = −0.87, *p* < 0.05) between the proximity estimates (Table 4) and the respective mean per road type (Table 1) proves their model integration plausible. Nevertheless, the mapped residuals and the standard deviations shown in Table 1 reveal local constraints when relying on six different functional road types only. Additional information, if available, such as driving speed, traffic volume, and road surface could help declaring these variations. Further, also the urban morphology could be represented in further detail. This could be achieved by invoking shape and landscape metrics. However, alike variables tend to also correlate, such as the linear model may be biased. This is very well exemplified with the cumulated length of residential roads within a radius of 800 m and the land-cover fraction of artificial land at the same radius (*r* = 0.795, *p* < 0.001). Last, when selecting the most relevant contextual features, the rather generous radii of the moving windows were surprising. Particular, a radius of 800 m for the TPI of urban morphology does not correspond to the small-scale acoustical phenomena caused by built-up morphology [38] and might have led to insignificant estimates. We assume that the large proportions of sparsely built-up areas were grasped as a major spurious collinearity instead of a local detail by the overall linear feature selector. We thus apprehend, selecting features based on univariate regression models as suggested by Ragettli et al. [22] and Liu et al. [26] cannot meet the multifactorial relationships observed. Second, with respect to the observed residuals, the linear regression was not capable of coping with acoustical phenomena such as reflections, refractions, and shadowing effects. While this could be encountered by a larger and more complex feature space, more elaborate models capable of grasping higher dimensional interactions need to be considered in the future. First steps in this direction were presented by Liu et al. [26] comparing random forest regressions against generalized additive models. Considering ongoing developments in computer sciences, deep convolutional neural networks hold immense potential. In the combination of the aspect mentioned above, future works shall investigate models more robust in regards to collinearity, such as they are capable of considering multiple contextual scales altogether. The deployment of a linear model here, however, was important to investigate the sampling schemes and their consequential artefacts.

The resulting broad scale noise assessment is novel for most of the small communities in the Rhine valley and fills a previously blind spot spatially. Further, congruent to our continuous base data, the predicted map depicts local variances below of the critical mapping threshold of 55 dB(A) and therefore enables analysis of environmental justice in higher granularity for the suburban parts in the Rhine valley as well. The subsequent residual analyses revealed that the most common noise levels between 35 and 75 dB(A) *L*_den_ were estimated concurrent to the conventional END conform map. With respect to exposure sciences, it is important to note that overall the map lacks accuracy for very loud and quite areas, respectively. In addition, the error was found to be lower for built-up areas in particular.

## Conclusion

In general, noise is a complex phenomenon, consisting of emission, propagation, and interactions with the environment. Therefore, the—very accurate—engineering noise mapping methods are highly sophisticated (see [14]) but technically inapplicable for large-scale epidemiological studies [21]. In this study we showed, however, that the acoustical phenomena being encoded in such END conform maps can be used to train statistical models, also known as LURs. Subsequent extrapolations can then be applied to estimate noise accurately at comparably low costs.

Yet, utilizing this most valuable data source, we were able to show that sampling design has a major impact on such models. Regarding the models’ predictive power, we conclude that the decreasing overall *R*^2^ at larger sample sizes reflects the complexity of acoustical phenomena. In few cases, smaller sample sizes could grasp them, but most probably lead to oversimplifying these physical mechanisms. This effect was most visible observed at *N* = 50 and LGOCV_50%_, where only 25 points were used to carry the regression, but dramatically underperformed during the validation. Vice versa, due to spatial autocorrelation, very large sample sizes compromise conventional leave-*n*-out cross-validation approaches. Following the guide by Roberts et al. [28], we recommend spatially blocked cross-validations. However, as each cross-validation approach tests distinct transfer abilities, we conclude the combined evaluation of multiple CV approaches being most meaningful. Last but not least, as to the unmet discrepancy between END conform noise maps and the reproductions using LUR, we acknowledge further development possibilities related to feature space and model architectures. Eventually, the aim must be to enable large-scale noise pollution assessments of previously excluded areas. Only then, affected populations can be identified and subsequent noise attenuation measures may be taken.

## Supplementary information

Supplementary material
