# Supplementary material for: Predicting traffic noise using land-use regression—a scalable approach
Source: J Expo Sci Environ Epidemiol. 2021 Jul 2;32(2):232–43. doi: 10.1038/s41370-021-00355-z (PMC8920888; doi:10.1038/s41370-021-00355-z)

Predicting traffic noise using land use regression – A scalable approach

Jeroen Staab, Arthur Schady, Matthias Weigand, Tobia Lakes, Hannes Taubenböck

Journal Of Exposure Science And Environmental Epidemiology; 2021

# Supplementary Material

## Tables

Table S1: Review of studies investigating urban noise using land use regression models. Hashtags denote constraints considered for stratified sample localization. LM = Multivariate Linear Model, GAM = Generalized Additive Model, RF = Random Forest, whereas a suffix of + or – corresponds to forwards, respectively backwards feature selection.

|  | Area of interest |  | Sampling | |  | Model | | | |  | | Cross-validation | | | |  | | Final model | | |
| --- | --- | --- | --- | --- | --- | --- | --- | --- | --- | --- | --- | --- | --- | --- | --- | --- | --- | --- | --- | --- |
|  |  |  |  | |  |  | |  | |  |  |  | | | |  |  |  | | |
|  |  |  | Number of sampling sites | Scheme |  | Model | Number of selected predictors | |  | | Method | | RMSE | R² |  | | adjusted R² | | Prediction |  |
| Xie et al., (2011) | Dalian, China. City + Metropolitan Area |  | 202 | Stratified  (#Land-use) |  | LM- | 4 | |  | | LGOCV_50%_ | | - | - |  | | - | | 200x200m |  |
| Goudreau et al., (2014) | Montreal, Canada. 500km² |  | Summer = 87 Winter = 62 | Stratified  (#Traffic  #Population) |  | GAM- | Summer = 11 Winter = 12 | |  | | LOOCV | | Summer = 3.3 dB(A) Winter = 4.5 dB(A) | - |  | | Summer = 0.64 Winter = 0.40 | | 20x20m |  |
| Aguilera et al., (2015) | Three mid-sized European cities |  | Basel = 60 Girona = 40 Grenoble = 41 | Stratified  (#Traffic  #Land-cover  #Population) |  | LM+ | Basel = 3 Girona = 4 Grenoble = 2 | |  | | LOOCV | | GIS Model Basel = 3.81 dB(A) Girona = 2.47 dB(A) Grenoble = 3.76 dB(A) Best Model Basel = 3.65 dB(A) Girona = 2.25 dB(A) Grenoble = 3.76 dB(A) | GIS Model Basel = 0.66 Girona = 0.84 Grenoble = 0.69 Best Model Basel = 0.68 Girona = 0.88 Grenoble = 0.69 |  | | Sequential R²! GIS Model Basel = 0.66 Girona = 0.87 Grenoble = 0.73 Best Model Basel = 0.70 Girona = 0.89 Grenoble = 0.73 | | 50x50m |  |
| Ragettli et al., (2016) | Montreal, Canada |  | In 2010 = 87 In 2014 = 117 Both = 204 | Stratified  (#Traffic  #Population) |  | GAM+ | LAeq24 = 16 Lnight = 14 Lden =16 | |  | | LOOCV | | LAeq24 = 3.54 Lnight = 3.97 Lden =3.71 | - |  | | LAeq24 = 0.68  Lnight = 0.59  Lden 0.69 | | 20x20m |  |
| Sieber et al., (2017) | Western Cape, South Africa. Informal Settlements |  | 134 | Stratified  (#Air-pollution) |  | LM+ | 5 | |  | | No CV | | - | - |  | | Overall = 0.13 | | - |  |
| Harouvi et al., (2018) | Two/Three cities in Israel |  | TVL, Tel Aviv = 99 BS, Beer Sheva = 71 | Random |  | LM- | 12 | |  | | LGOCV_10%_ | | Rush Hour TVL = 2.87 dB(A) BS = 4.33 dB(A) Off Peak TVL = 2.84 dB(A) BS = 4.66 dB(A) | Rush Hour TVL = 0.79 BS = 0.52 Off Peak TVL = 0.73 BS = 0.32 |  | | Rush Hour TVL = 0.83 BS = 0.57 TVL->Bat Yam = 0.93 Off Peak TVL = 0.82 BS = 0.42 | | 100x100m |  |
| Chang et al., (2019) | Taichung, Taiwan |  | 50 | Stratified  (#Traffic  #Land-use) |  | LM+ | 6 | |  | | LOOCV | | Leq24h = 2.09 Lnight = 2.80 | Leq24h = 0.77 Lnight = 0.72 |  | | Leq24h = 0.80 Lnight = 0.76 | | - |  |
| Liu et al., (2020) | Five large Canadian Cities |  | Montreal = 204, Toronto = 271, Halifax = 48, Longueil = 104 TOTAL = 729 | Stratified  (#Traffic  #Land-use  #Population) |  | GAM- RM | Global Model GAM = 25 RF = 33 | |  | | LGOCV_50%_ LGOCV_25%_ LGOCV_10%_ LOOCV | | Global Model CV50% GAM = 4.97 dB(A) RF = 4.62 dB(A) Global Model CVone GAM = 4.99 dB(A) RF = 4.44 dB(A) | - |  | | Global Model CV50% GAM = 0.48 RF = 0.55 Global Model CVon GAM = 0.47 RF = 0.58 | | postal-code-level |  |

Table S2: Tabular summary of road L_den_ in Coblenz and its 30 statistical districts. Area-fraction (AF) refers to percentage of area covered by this class.

| Statistical District | | AF [%] | L_den_ | | |  |
| --- | --- | --- | --- | --- | --- | --- |
| Code | Name |  | Min. | Mean (sd) | Max. | |
| Are. | Arenberg | 6.12 | 22.20 | 46.15 (9.02) | 79.10 | |
| I. | Immendorf | 2.43 | 10.30 | 38.34 (9.52) | 70.40 | |
| Arz. | Arzheim | 4.09 | 17.80 | 48.55 (10.52) | 80.10 | |
| Ke. | Kesselheim | 5.71 | 38.20 | 59.09 (7.84) | 88.80 | |
| B. | Bubenheim | 3.12 | 43.00 | 60.86 (8.42) | 87.80 | |
| Mo. | Moselweiß | 1.95 | 37.60 | 53.94 (6.83) | 74.60 | |
| La. | Lay | 2.42 | 27.70 | 49.04 (6.69) | 70.60 | |
| St. | Stolzenfels | 2.46 | 16.70 | 49.8 (11.35) | 79.30 | |
| W. | Wallersheim | 1.78 | 37.00 | 51.3 (4.84) | 71.00 | |
| Ne. | Neuendorf | 1.38 | 35.90 | 55.63 (5.77) | 79.30 | |
| LÃ¼. | Lützel | 3.3 | 39.70 | 59.66 (8.07) | 85.50 | |
| Me. | Metternich | 4.49 | 24.90 | 52.71 (6.86) | 77.20 | |
| Ra. | Rauental | 1.74 | 35.30 | 56.13 (7.98) | 83.60 | |
| Alt. | Altstadt | 1.2 | 34.50 | 55.05 (7.89) | 77.30 | |
| Go. | Goldgrube | 1.05 | 41.00 | 53.94 (7.87) | 80.80 | |
| O. | Oberwerth | 1.21 | 44.40 | 56.99 (7.22) | 79.00 | |
| Sü. | Süd | 1.37 | 34.60 | 58.98 (8.4) | 80.10 | |
| Ni. | Niederberg | 1.87 | 29.10 | 47.58 (9.77) | 82.50 | |
| E. | Ehrenbreitstein | 1.61 | 18.20 | 46.29 (11.47) | 80.00 | |
| Ho.H. | Horchheimer Höhe | 7.17 | 0.50 | 40.45 (12.46) | 81.20 | |
| Ho. | Horchheim | 1.15 | 40.30 | 58.22 (6.87) | 81.70 | |
| Ka.G. | Karthäuserhofgelände | 16.41 | 9.40 | 45.65 (10.95) | 80.60 | |
| Ka.F. | Karthause Flugfeld | 0.82 | 32.40 | 51.31 (8.39) | 72.80 | |
| Ka.N. | Karthause Nord | 0.79 | 32.10 | 46.41 (7.92) | 80.10 | |
| Rü. | Rübenach | 12.26 | 38.80 | 59.41 (8.31) | 89.10 | |
| Gü. | Güls | 7.82 | 26.60 | 49.12 (6.93) | 76.50 | |
| As. | Asterstein | 1.6 | 31.20 | 46.81 (7.11) | 80.70 | |
| Mi. | Mitte | 0.57 | 40.40 | 56.72 (7.92) | 77.20 | |
| Pf. | Pfaffendorf | 1 | 37.90 | 54.93 (7.8) | 81.00 | |
| Pf.H. | Pfaffendorfer Höhe | 1.1 | 34.20 | 53.83 (8.01) | 80.80 | |

Table S3: Tabular summary of road L_den_ in Coblenz, disaggregated into urban atlas classes. Area-fraction (AF) refers to percentage of area covered by this class.

| Urban Atlas | | AF [%] | L_den_ | | |
| --- | --- | --- | --- | --- | --- |
| Code | Class |  | Min. | Mean (sd) | Max. |
| 11100 | Continuous Urban fabric (S.L. > 80%) | 2.07 | 30.90 | 52.18 (7.68) | 81.40 |
| 11210 | Discontinuous Dense Urban Fabric (S.L.: 50% - 80%) | 8.7 | 18.20 | 50.57 (7.58) | 79.80 |
| 11220 | Discontinuous Medium Density Urban Fabric (S.L.: 30%, 50%) | 2.97 | 20.30 | 49.84 (8.01) | 80.50 |
| 11230 | Discontinuous Low Density Urban Fabric (S.L.: 10% - 30%) | 0.36 | 25.30 | 43.41 (7.25) | 71.50 |
| 11240 | Discontinuous very low density urban fabric (S.L. &l, 10%) | 0.03 | 39.60 | 49.2 (2.9) | 57.70 |
| 11300 | Isolated Structures | 0.25 | 16.10 | 46.74 (11.69) | 74.40 |
| 12100 | Industrial, commercial, public, military and private units | 10.84 | 29.00 | 54.58 (8.6) | 85.50 |
| 12210 | Fast transit roads and associated land | 0.62 | 66.50 | 81.29 (5.72) | 89.10 |
| 12220 | Other roads and associated land | 5.56 | 10.60 | 59.61 (13.23) | 87.00 |
| 12230 | Railways and associated land | 0.91 | 38.90 | 60.81 (7.3) | 82.00 |
| 12300 | Port areas | 0.18 | 44.50 | 49.19 (1.46) | 53.00 |
| 12400 | Airports | 0.06 | 45.20 | 47.16 (0.43) | 48.20 |
| 13100 | Mineral extraction and dump sites | 0.22 | 19.70 | 43.09 (11.72) | 71.70 |
| 13300 | Construction sites | 0.13 | 49.20 | 56.16 (4.12) | 71.70 |
| 13400 | Land without current use | 0.33 | 39.70 | 54.83 (5.95) | 77.00 |
| 14100 | Green urban areas | 3.25 | 34.10 | 54.74 (7.3) | 84.20 |
| 14200 | Sports and leisure facilities | 1.69 | 18.30 | 53.19 (6.86) | 79.20 |
| 21000 | Arable land (annual crops) | 13.16 | 19.20 | 56.73 (8.94) | 88.40 |
| 23000 | Pastures | 10.2 | 11.40 | 49.33 (10.02) | 86.80 |
| 31000 | Forests | 32.2 | 0.50 | 45 (11.14) | 85.90 |
| 32000 | Herbaceous vegetation associations | 1.22 | 20.90 | 47.72 (10.4) | 77.90 |

Table S4: Summary of R² replicated using 100 seeds, aggregated by sampling scheme and – size.

|  |  | R² | | |  | RMSE | | |  | MAE | | |
| --- | --- | --- | --- | --- | --- | --- | --- | --- | --- | --- | --- | --- |
| Sampling | N | q5% | mean (sd) | q95% |  | q5% | mean (sd) | q95% |  | q5% | mean (sd) | q95% |
| Random | 50 | 0.75 | 0.83 (0.05) | 0.9 |  | 3.02 | 4.44 (0.88) | 5.73 |  | 2.3 | 3.4 (0.65) | 4.27 |
|  | 100 | 0.67 | 0.75 (0.04) | 0.82 |  | 4.51 | 5.43 (0.64) | 6.49 |  | 3.33 | 4.07 (0.49) | 4.82 |
|  | 200 | 0.65 | 0.71 (0.03) | 0.76 |  | 5.19 | 5.97 (0.48) | 6.7 |  | 3.92 | 4.4 (0.35) | 4.99 |
|  | 500 | 0.65 | 0.68 (0.02) | 0.72 |  | 5.76 | 6.26 (0.32) | 6.81 |  | 4.2 | 4.54 (0.23) | 4.91 |
|  | 1000 | 0.65 | 0.67 (0.02) | 0.7 |  | 5.97 | 6.33 (0.23) | 6.73 |  | 4.33 | 4.58 (0.17) | 4.88 |
| Systematic | 50 | 0.69 | 0.79 (0.06) | 0.87 |  | 3.66 | 5.03 (0.94) | 6.43 |  | 2.74 | 3.81 (0.72) | 4.79 |
|  | 100 | 0.67 | 0.73 (0.04) | 0.78 |  | 4.98 | 5.87 (0.6) | 6.85 |  | 3.76 | 4.41 (0.43) | 4.99 |
|  | 200 | 0.66 | 0.7 (0.03) | 0.74 |  | 5.53 | 6.12 (0.37) | 6.66 |  | 4.15 | 4.52 (0.23) | 4.85 |
|  | 500 | 0.65 | 0.68 (0.02) | 0.71 |  | 5.97 | 6.34 (0.24) | 6.7 |  | 4.39 | 4.6 (0.16) | 4.88 |
|  | 1000 | 0.66 | 0.67 (0.01) | 0.69 |  | 6.08 | 6.34 (0.16) | 6.57 |  | 4.42 | 4.58 (0.09) | 4.72 |
| Stratified_Lden_ | 50 | 0.82 | 0.88 (0.04) | 0.93 |  | 4.4 | 6.25 (1.16) | 8.16 |  | 3.53 | 4.88 (0.9) | 6.51 |
|  | 100 | 0.8 | 0.83 (0.03) | 0.89 |  | 5.97 | 7.31 (0.7) | 8.28 |  | 4.78 | 5.68 (0.54) | 6.44 |
|  | 200 | 0.77 | 0.81 (0.02) | 0.84 |  | 7.14 | 7.89 (0.47) | 8.56 |  | 5.41 | 6.08 (0.37) | 6.71 |
|  | 500 | 0.77 | 0.79 (0.02) | 0.81 |  | 7.72 | 8.27 (0.35) | 8.8 |  | 5.98 | 6.4 (0.26) | 6.77 |
|  | 1000 | 0.77 | 0.78 (0.01) | 0.8 |  | 8.02 | 8.35 (0.23) | 8.74 |  | 6.2 | 6.44 (0.16) | 6.72 |
| Stratified_Urb.Atl._ | 50 | 0.81 | 0.88 (0.04) | 0.94 |  | 2.81 | 3.86 (0.74) | 5.32 |  | 2.18 | 2.97 (0.55) | 3.99 |
|  | 100 | 0.73 | 0.81 (0.04) | 0.87 |  | 3.94 | 5.02 (0.67) | 6.22 |  | 3.07 | 3.74 (0.45) | 4.49 |
|  | 200 | 0.72 | 0.77 (0.03) | 0.81 |  | 4.92 | 5.64 (0.44) | 6.51 |  | 3.58 | 4.1 (0.34) | 4.76 |
|  | 500 | 0.7 | 0.74 (0.02) | 0.77 |  | 5.45 | 5.94 (0.31) | 6.48 |  | 3.84 | 4.22 (0.23) | 4.67 |
|  | 1000 | 0.71 | 0.74 (0.02) | 0.76 |  | 5.67 | 5.97 (0.2) | 6.28 |  | 4.01 | 4.24 (0.14) | 4.44 |

## Figures

Figure S1: Map showing Coblenz, located in Rhineland-Palatinate. White lines separate administrative units, where solid lines depict NUTS-3 (gadm.org) and dotted lines the 30 statistical units of Coblenz (openstreetmaps.org, abbreviations are decoded in appendix 2). Grey lines represent road infrastructure, itemized by road type (openstreetmaps.org). Bright red polygons represent built-up areas (BKG), while the natural environment (Weigand et al., 2020) together with a hill-shade effect (EU-DEM) represent the natural environment.


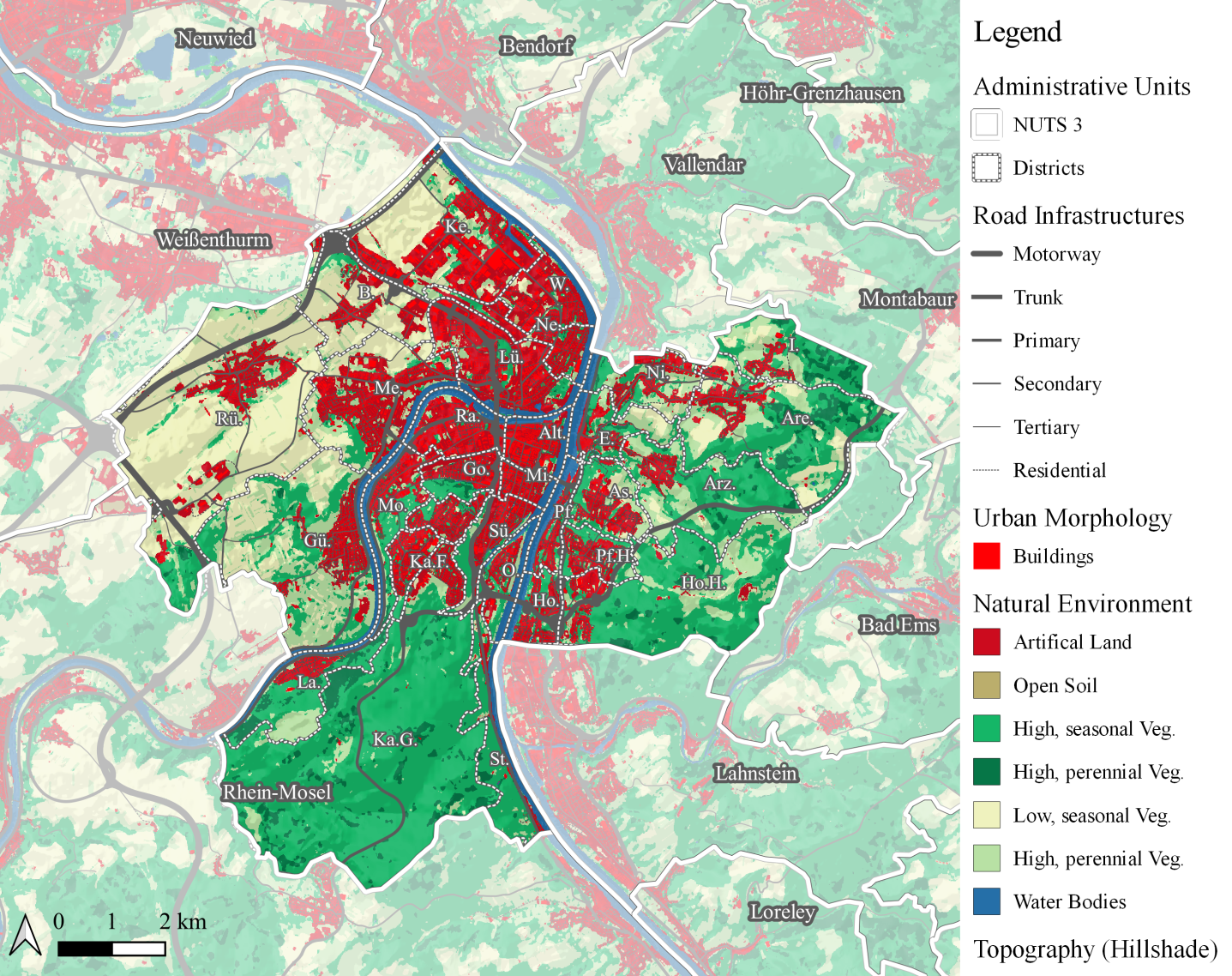


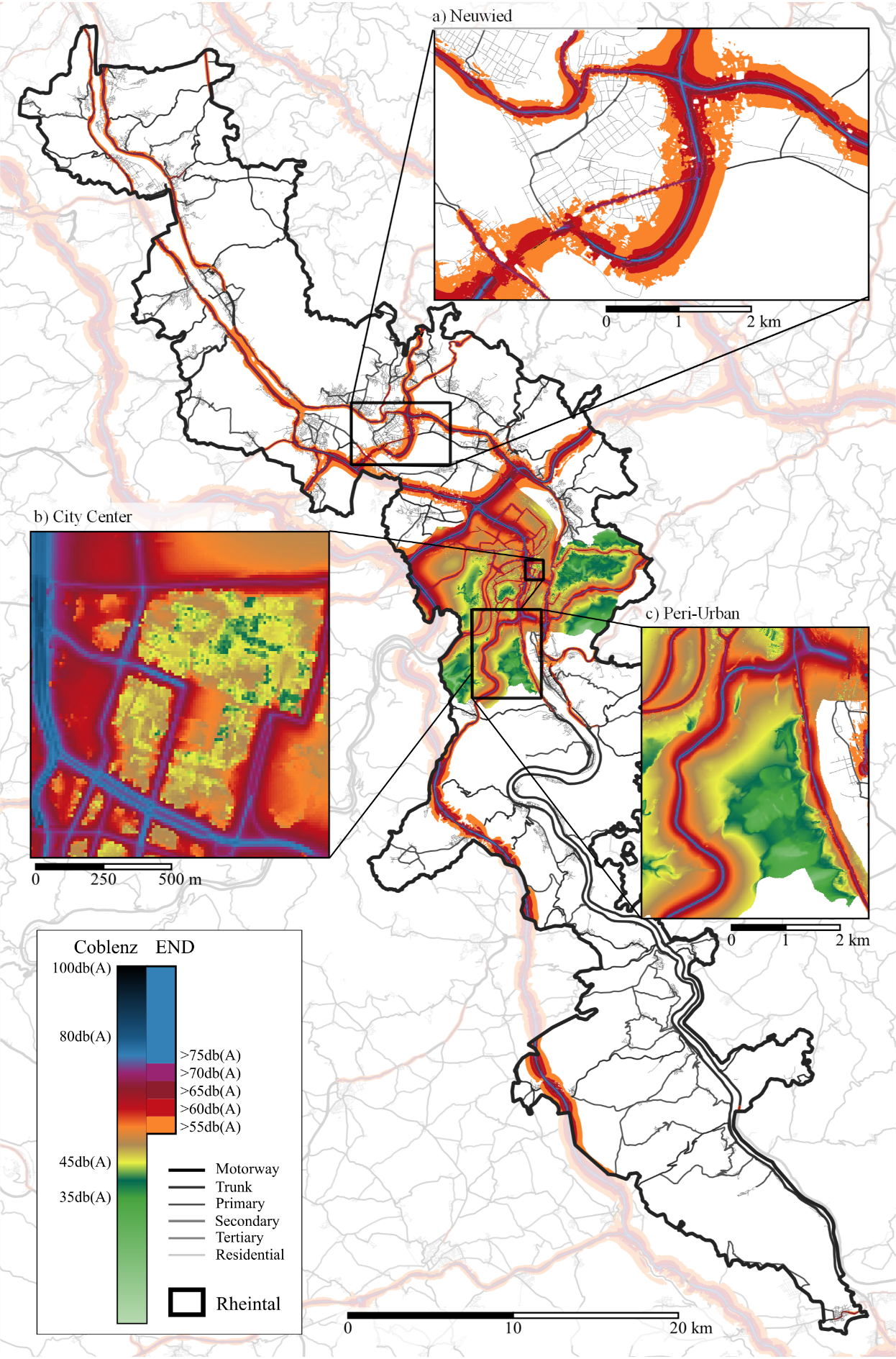
Figure S2: Visualization of noise simulations for the city of Coblenz 2017 and END conform map for its surroundings. Additional zooms provide insights on small scale acoustical effects in the historic center (Altstadt, b) and along a primary road (B327, c). Continuous color scheme akin to DIN 18005.

Figure S3: Mapped residuals a). Negative values are colored red and represent areas being mapped “too loud”, while positive values (blue) are “too quiet”. The supplementary histogram b) depicts the respectively covered areas. The subset of build-up areas is outlined black. Boxplots c) summarize residuals in regards to predicted noise levels.


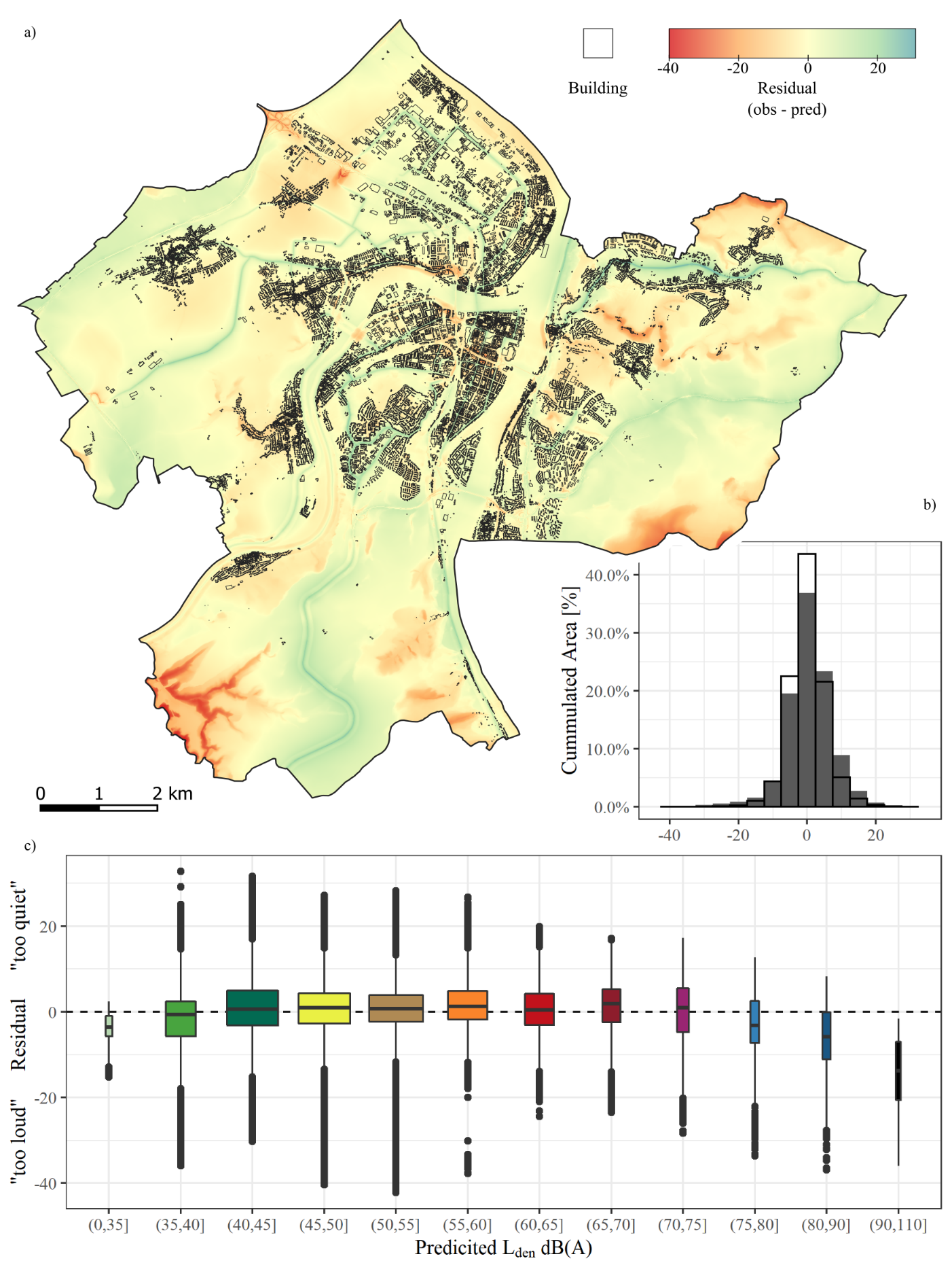

Supplement: Supplementary file 1 — Supplementary material [file 41370_2021_355_MOESM1_ESM.docx]
